# Pre-Transplant Prognostic Nutritional Index Independently Predicts Progression-Free Survival After Autologous Stem Cell Transplantation in Lymphoma

**DOI:** 10.3390/jcm15093549

**Published:** 2026-05-06

**Authors:** Hüseyin Atacan, Volkan Aslan, Alper Topal, Nurlan Mammadzada, Gizem Yıldırım, Gökçe Gül Güneysu, Berkan Karadurmuş, Esmanur Kaplan Tüzün, Ömer Faruk Kuzu, Efe Cem Erdat, Musa Barış Aykan, İsmail Ertürk, Nuri Karadurmuş

**Affiliations:** 1 Department of Medical Oncology, Gülhane Training and Research Hospital, University of Health Sciences, Ankara 06010, Türkiye; dr.volcanaslan@gmail.com (V.A.); mammadzadanurlan@gmail.com (N.M.); gizemyildirim_@hotmail.com (G.Y.); gokcegulguneysu@gmail.com (G.G.G.); berkankaradurmus@gmail.com (B.K.); esmanurkaplan@hotmail.com (E.K.T.); musabarisaykan@gmail.com (M.B.A.); ierturk@hotmail.com (İ.E.); drnkaradurmus@yahoo.com (N.K.); 2 Department of Medical Oncology, Tokat Gaziosmanpaşa University, Tokat 60150, Türkiye; dralpertopal@gmail.com; 3 Department of Medical Oncology, Çankırı State Hospital, Çankırı 18100, Türkiye; drfarukkuzu@gmail.com

**Keywords:** autologous stem cell transplantation, lymphoma, prognostic nutritional index, progression-free survival, pre-transplant response, CD34^+^ cell dose

## Abstract

**Background:** Autologous stem cell transplantation (ASCT) is a standard treatment for relapsed or high-risk lymphoma. While disease-related factors are well-known, the impact of host-related factors like nutritional status remains less defined. We aimed to evaluate the prognostic value of the prognostic nutritional index (PNI) and other factors in lymphoma patients undergoing ASCT. **Methods:** We conducted a single-center retrospective cohort study including adult patients with Hodgkin and non-Hodgkin lymphoma who underwent ASCT between January 2015 and December 2023. Pre-transplant clinical, laboratory, and transplant-related variables were analyzed. The prognostic nutritional index (PNI) was calculated using serum albumin and absolute lymphocyte count and dichotomized according to the cohort median. Progression-free survival (PFS) and overall survival (OS) were evaluated using Kaplan–Meier and Cox regression analyses. We conducted a retrospective single-center cohort study including adult patients with Hodgkin and non-Hodgkin lymphoma who underwent ASCT between January 2015 and December 2023. **Results:** A total of 43 patients were included. Median age was 38 years, and 72.1% were male. Patients transplanted in complete remission (CR) had significantly longer PFS compared with those transplanted in partial remission (PR) (log-rank *p* = 0.022). Patients with higher pre-ASCT PNI demonstrated significantly improved PFS (median 45 vs. 7 months; log-rank *p* = 0.021). In multivariable Cox regression analysis, both higher PNI (HR 0.39; 95% CI 0.16–0.97; *p* = 0.043) and complete remission prior to ASCT (HR 0.41; 95% CI 0.17–0.98; *p* = 0.046) remained independently associated with improved PFS. Higher infused CD34^+^ (hematopoietic stem cell) dose was associated with shorter hospitalization but showed no statistically significant association with engraftment kinetics or survival. No variable was independently associated with OS, likely due to the limited number of death events. **Conclusions:** Pre-transplant prognostic nutritional index and disease response independently predict progression-free survival after ASCT in lymphoma. These findings highlight the complementary role of host-related and disease-related factors in transplant outcomes and suggest that PNI may serve as a practical tool for pre-transplant risk stratification and patient optimization. Given the small sample size and limited number of events, these findings should be interpreted with caution.

## 1. Introduction

Autologous stem cell transplantation (ASCT) remains a cornerstone in the treatment for patients with relapsed or high-risk lymphoma and is widely used as consolidation therapy in both Hodgkin and non-Hodgkin lymphoma [1,2,3]. Despite substantial advances in induction regimens, supportive care, and transplant techniques, post-transplant outcomes remain heterogeneous, and disease relapse continues to represent the leading cause of treatment failure following ASCT [2,4,5].

Traditionally, prognostic assessment in lymphoma patients undergoing ASCT has focused primarily on disease-related factors, including histological subtype, chemosensitivity, and established prognostic indices such as the International Prognostic Index (IPI) [6]. Among these, pre-transplant disease status—particularly the achievement of complete remission prior to ASCT—has consistently been shown to be a strong determinant of post-transplant progression-free survival (PFS) [1,2,5]. Functional imaging-based response assessment using FDG-PET has further refined risk stratification, demonstrating superior outcomes in patients achieving metabolic complete remission before transplantation [5,7]. However, disease-specific parameters alone may not fully capture the complexity of post-transplant outcomes, particularly in real-world patient populations.

In recent years, increasing attention has been directed toward host-related factors, including nutritional status, systemic inflammation, and immune competence, as potential modulators of treatment tolerance and long-term disease control [8,9,10,11]. The prognostic nutritional index (PNI), calculated using serum albumin level and absolute lymphocyte count, represents a simple and readily available composite marker reflecting both nutritional reserve and immune function [12]. Hypoalbuminemia has been associated with systemic inflammation and adverse oncologic outcomes, while lymphopenia reflects impaired cellular immunity and reduced anti-tumor immune surveillance [8,9,10,11].

PNI has been shown to predict survival outcomes across a broad range of solid tumors and hematologic malignancies and has demonstrated prognostic value in patients with lymphoma treated with conventional chemotherapy regimens [13,14,15]. Moreover, in the hematopoietic stem cell transplantation setting—particularly in allogeneic transplantation—lower pre-transplant PNI has been associated with increased transplant-related complications, higher non-relapse mortality, and inferior survival outcomes [16]. However, data regarding the prognostic significance of PNI in patients undergoing ASCT for lymphoma remain limited.

In addition to disease- and host-related factors, transplant-related parameters such as infused CD34^+^ cell dose may influence early post-transplant outcomes. Higher CD34^+^ cell doses have been associated with faster hematopoietic recovery, reduced infectious complications, and decreased supportive care requirements [17,18,19,20]. Nevertheless, the impact of CD34^+^ cell dose on clinical outcomes beyond engraftment kinetics, including survival endpoints, remains incompletely defined.

Given these considerations, we conducted a single-center retrospective study to evaluate the impact of pre-transplant disease status, nutritional and inflammatory markers—including PNI—and transplant-related factors on early transplant outcomes and survival in patients with lymphoma undergoing ASCT. Our aim was to identify clinically relevant predictors of progression-free and overall survival that may complement existing prognostic frameworks and inform pre-transplant risk stratification and patient optimization strategies. To our knowledge, evidence integrating nutritional status, treatment response, and transplant-related parameters in a single real-world ASCT lymphoma cohort remains limited.

## 2. Materials and Methods

### 2.1. Study Design and Patient Population

This single-center retrospective cohort study included consecutive adult patients with lymphoma who underwent autologous stem cell transplantation (ASCT) at Gulhane Training and Research Hospital between January 2015 and December 2023. Follow-up data were collected through 1 May 2025, which was defined as the data cut-off date. Patients with Hodgkin lymphoma (HL) or non-Hodgkin lymphoma (NHL) who received ASCT as part of standard clinical practice were eligible for inclusion. All eligible patients were included in the study cohort. Analyses were performed on an available-case basis; patients with missing data for specific variables (e.g., progression status) were excluded only from the corresponding analyses.

The study was conducted in accordance with the Declaration of Helsinki and was approved by the local institutional ethics committee. Due to the retrospective nature of the study, the requirement for informed consent was waived.

### 2.2. Data Collection

Clinical, laboratory, and transplant-related data were retrieved from electronic medical records. Baseline variables included age, sex, lymphoma subtype, disease stage at diagnosis, IPI score at diagnosis (for applicable subtypes), prior treatment lines, and disease status prior to ASCT. Pre-ASCT response was categorized as complete remission (CR) or partial remission (PR) based on radiologic and/or metabolic assessment according to institutional standards.

The International Prognostic Index (IPI) was calculated based on standard clinical parameters at diagnosis, including age, disease stage, serum LDH level, performance status, and extranodal involvement. The International Prognostic Score (IPS) was not calculated due to a lack of uniformly recorded component variables at a consistent clinical time point. Disease stage was documented at diagnosis, whereas performance status and laboratory parameters were primarily available from the pre-transplant period.

Laboratory parameters obtained prior to conditioning included white blood cell count, absolute neutrophil and lymphocyte counts, hemoglobin, platelet count, serum albumin, creatinine, lactate dehydrogenase (LDH), and inflammatory markers where available. The prognostic nutritional index (PNI) was calculated using the following formula [12]:

PNI = 10 × serum albumin (g/dL) + 0.005 × absolute lymphocyte count (/mm^3^)

In the absence of a universally accepted cutoff value and given the relatively small sample size, PNI was dichotomized using the cohort median value (47.95) to avoid arbitrary threshold selection and ensure balanced group sizes in this exploratory analysis.

### 2.3. Transplant Procedure and Supportive Care

All patients underwent ASCT in the relapsed or refractory setting. Stem cell mobilization and collection were performed according to institutional protocols. Stem cell mobilization was performed using granulocyte colony-stimulating factor (G-CSF) alone. Plerixafor was administered to selected patients with inadequate mobilization. High-dose chemotherapy was administered as part of the conditioning regimen following stem cell collection. Pre-transplant clinical and laboratory parameters were defined as those obtained immediately prior to the initiation of the conditioning regimen.

The target CD34^+^ cell dose was defined as ≥2 × 10^6^ cells/kg. Conditioning regimens were selected based on lymphoma subtype and institutional practice, with BEAM being the most commonly used regimen. The infused CD34^+^ cell dose was recorded for each patient and analyzed both as a continuous variable and dichotomized according to the cohort median.

Supportive care, including antimicrobial prophylaxis, transfusion support, and granulocyte colony-stimulating factor (G-CSF) administration, was provided according to standard institutional guidelines. Missing data were handled using a complete-case analysis approach, and no imputation was performed.

### 2.4. Response Assessment

Pre-transplant treatment response was assessed uniformly in all patients using positron emission tomography–computed tomography (PET-CT) according to the Lugano 2014 classification. Metabolic response was evaluated using the Deauville 5-point scale. Complete response (CR) was defined as a Deauville score of 1–3, while partial response (PR) was defined as a Deauville score of 4 with residual uptake above background but reduced compared with baseline.

All patients underwent PET-CT–based response assessment prior to ASCT as part of routine clinical care. PET-CT examinations were interpreted by experienced nuclear medicine physicians at our institution.

### 2.5. Study Endpoints and Definitions

Neutrophil engraftment was defined as the first of three consecutive days with an absolute neutrophil count ≥500/µL. Platelet engraftment was defined as the first of seven consecutive days with a platelet count ≥20,000/µL without transfusion support. Febrile neutropenia, documented infections, veno-occlusive disease, intensive care unit admission, and 100-day transplant-related mortality were recorded.

**Figure 1:**
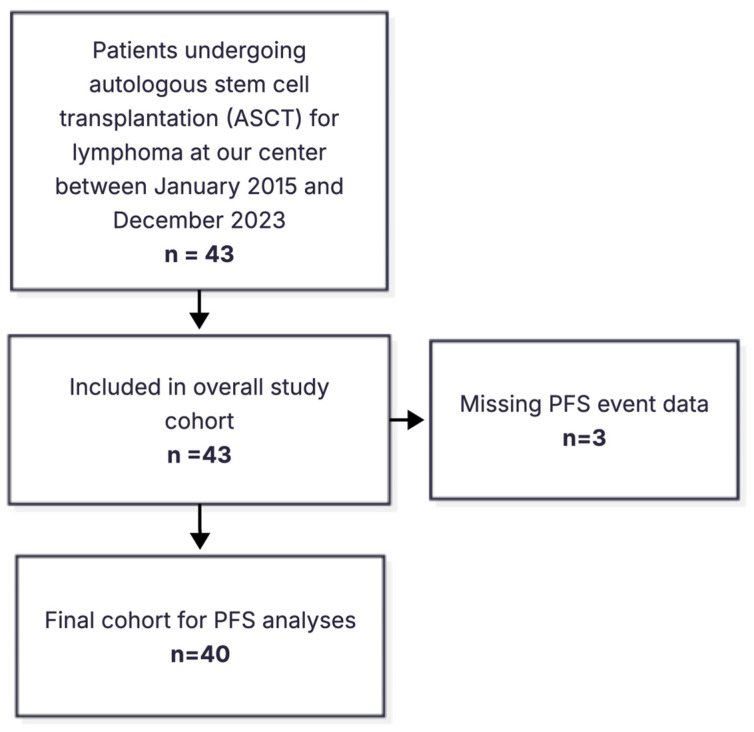
Study flow diagram of patient inclusion and progression-free survival analysis.

Progression-free survival (PFS) was defined as the time from ASCT to disease progression, relapse, or death from any cause. Patients with missing progression-free survival event data were excluded from response-based survival analyses. The study flow diagram for progression-free survival is shown in Figure 1. Overall survival (OS) was defined as the time from ASCT to death from any cause or last follow-up.

### 2.6. Statistical Analysis

Continuous variables were summarized using median and interquartile range (IQR), and categorical variables using frequencies and percentages. Comparisons between groups were performed using the Mann–Whitney U test or chi-square test, as appropriate. Median follow-up duration was calculated using the reverse Kaplan–Meier method. Survival outcomes were estimated using the Kaplan–Meier method and compared using the log-rank test. Kaplan–Meier curves were truncated at 60 months for PFS and 72 months for OS to avoid overinterpretation beyond time points with limited numbers at risk. Cox proportional hazards regression analyses were performed to identify factors associated with PFS and OS. For PFS analyses, 22 progression/death events occurred among 40 patients with complete PFS data, allowing a multivariable Cox model including two covariates, yielding an events-per-variable ratio of 11. In addition to categorical analyses based on median cut-off values, and to minimize information loss due to dichotomization, the prognostic nutritional index (PNI) was also analyzed as a continuous variable in Cox proportional hazards regression models to assess its association with progression-free and overall survival. Hazard ratios were calculated per unit increase in PNI. Sensitivity analyses were performed to assess the robustness of the findings across major histologic subgroups, including analyses restricted to Hodgkin lymphoma and diffuse large B-cell lymphoma. Variables considered for multivariable Cox regression analyses included those with established clinical relevance and/or a *p*-value < 0.10 in univariable analysis. Given the limited number of progression-free survival events, the final multivariable model was intentionally restricted to two covariates to minimize the risk of overfitting. A two-sided *p* value < 0.05 was considered statistically significant. All statistical analyses were performed using SPSS software (version 25; IBM Corp., Armonk, NY, USA).

## 3. Results

### 3.1. Baseline Characteristics

Baseline demographic and clinical characteristics of the study population are summarized in Table 1. The median age was 38 years (IQR 29), with a male predominance (72.1%). Histological subtypes included Hodgkin lymphoma (44.2%), diffuse large B-cell lymphoma (39.5%), mantle cell lymphoma (11.6%), and T-cell lymphoma (4.7%). At initial diagnosis, the majority of patients had advanced-stage disease; specifically, 40 patients (93%) were classified as Ann Arbor stage IV and 3 patients (7%) as stage III. The median body mass index (BMI) at the time of transplantation was 26 kg/m^2^ (IQR, 5). Nineteen patients (44.2%) had a BMI < 25 kg/m^2^, whereas 24 patients (55.8%) were categorized as overweight or obese (BMI ≥ 25 kg/m^2^). Among patients with DLBCL, the distribution of IPI scores at diagnosis was as follows: 24% low, 35% intermediate, and 41% high risk. Most patients (88.4%) had received one or two prior lines of therapy before ASCT. Regarding pre-transplant disease status, 53.5% of patients achieved a complete response (CR), while 46.5% had a partial response (PR). BEAM was the predominant conditioning regimen (90.7%).

### 3.2. Engraftment and Short-Term Outcomes

Median infused CD34^+^ cell dose was 3.6 (IQR 2.5) × 10^6^/kg. Neutrophil engraftment was successfully achieved in 42 patients (97.7%) at a median of 12 days (IQR: 1). Platelet engraftment also occurred in 42 patients (97.7%), with a median time of 14 days (IQR, 2.25). The median length of hospitalization was 28 days (IQR, 9). Notably, patients receiving higher CD34^+^ cell doses (≥3.6 ×10^6^/kg) experienced a significantly shorter hospital stay compared with those receiving lower doses (Mann–Whitney U test, *p* = 0.010). Although patients in the higher CD34^+^ dose group tended to achieve platelet engraftment earlier (13 [12–15] vs. 15 [14–17] days), this difference did not reach statistical significance (*p* = 0.113). Neutrophil engraftment time did not differ based on CD34^+^ cell dose (*p* = 1.00). Febrile neutropenia within the first 100 days post-ASCT occurred in 40 patients (93.0%). Documented infections were observed in 17 patients (39.5%). No cases of veno-occlusive disease were recorded. Intensive care unit admission was required in two patients (4.7%) and the 100-day transplant-related mortality (TRM) rate was 4.7% (n = 2). These early outcomes are summarized in Table 2.

### 3.3. Survival and Prognostic Factors

The median follow-up duration was 48 months (95% CI, 35–61 months). The median progression-free survival was 14 months (95% CI, 0–44.2), while the median overall survival (OS) was not reached at the time of analysis. BMI, when analyzed as a categorical variable, was not significantly associated with PFS (log-rank *p* = 0.568). Similarly, ECOG performance status (0 vs. 1) did not significantly impact PFS (median 40 vs. 27 months; log-rank *p* = 0.7), likely reflecting the relatively homogeneous and fit status of the transplant-eligible population.

PFS analysis according to pre-ASCT response was conducted in 40 patients with available response and follow-up data (22 CR and 18 PR). At the time of analysis, median overall survival had not been reached. In exploratory analyses stratified by histologic subtype, PFS did not differ significantly between patients with Hodgkin lymphoma and those with non-Hodgkin lymphoma (median PFS: 40 vs. 12 months; log-rank *p* = 0.746).

To ensure the robustness of our findings, sensitivity analyses were restricted to the two primary histologic subgroups (HL and DLBCL). These analyses confirmed that the pre-ASCT prognostic nutritional index (PNI) remained significantly associated with PFS, with effect estimates consistent with the primary analysis. This suggests that the prognostic value of PNI was independent of specific histologic subtypes. Furthermore, although PFS did not differ significantly across IPI risk groups at diagnosis (log-rank *p* = 0.610), this interpretation is limited by the small sample size in each category (low, n = 4; intermediate, n = 6; high, n = 7).

**Figure 2:**
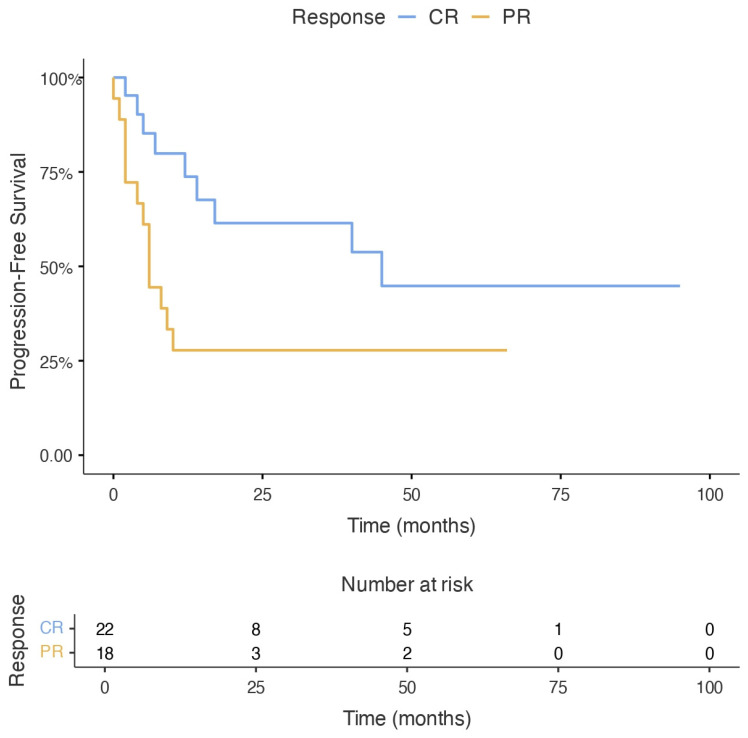
Kaplan–Meier curves for progression-free survival according to pre-ASCT response status. Patients transplanted in complete remission (CR) had significantly longer progression-free survival than those transplanted in partial remission (PR) (log-rank *p* = 0.022). Median progression-free survival was 45 months in the CR group and 6 months in the PR group. Hazard ratio for progression or death was 0.38 (95% CI, 0.18–0.82).

Notably, pre-ASCT response status emerged as a significant predictor of survival. Patients transplanted in CR demonstrated a markedly superior median PFS compared to those in PR (45 months [95% CI: 1.4–88.6] vs. 6 months [95% CI: 4.6–7.4]; log-rank *p* = 0.022). Univariable Cox regression analysis further substantiated this, showing that patients in CR had a significantly lower risk of progression or death (HR: 0.38, 95% CI: 0.18–0.82; *p* = 0.030) (Figure 2).

**Figure 3:**
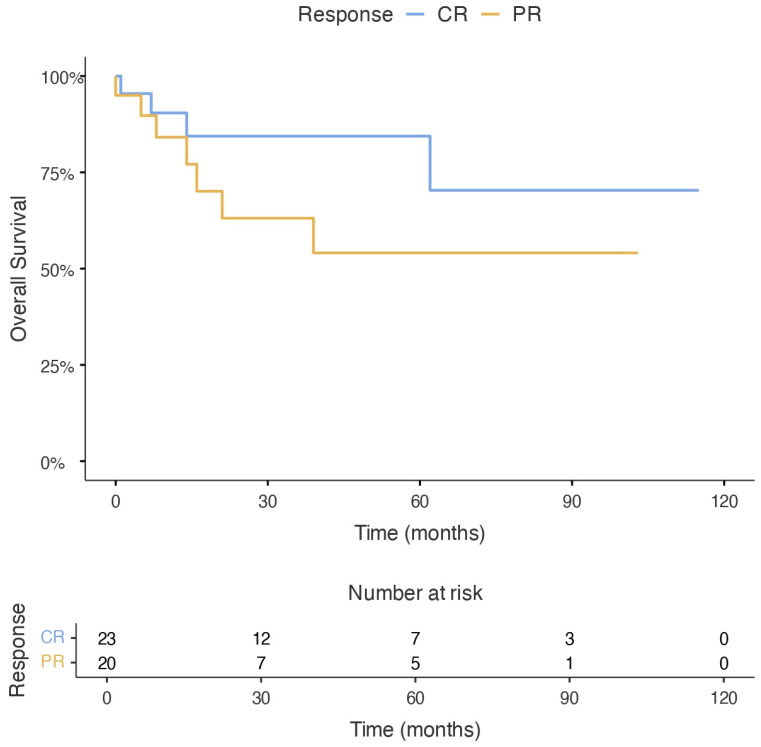
Kaplan–Meier curves for overall survival according to pre-ASCT response status (complete remission vs. partial remission). Patients transplanted in complete remission showed numerically improved overall survival compared with those transplanted in partial remission; however, the difference was not statistically significant (log-rank *p* = 0.188).

When patients were stratified according to pre-ASCT response status, those transplanted in complete remission showed numerically improved overall survival compared with patients transplanted in partial remission; however, this difference did not reach statistical significance (log-rank *p* = 0.188). The median overall survival was not reached in either group. Consistently, univariable Cox regression analysis showed that pre-ASCT CR was associated with a reduced risk of death, although this finding was not statistically significant (HR: 0.45; 95% CI: 0.13–1.53; *p* = 0.201) (Figure 3).

**Figure 4:**
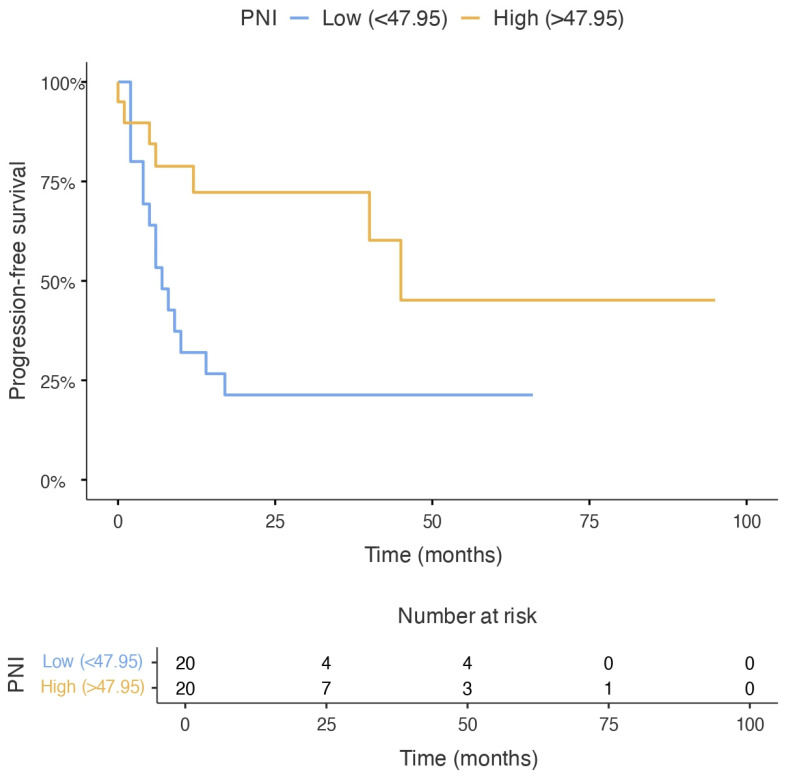
Kaplan–Meier curves for progression-free survival according to pre-transplant prognostic nutritional index (PNI). Patients with PNI ≥ 47.95 demonstrated significantly superior progression-free survival compared with those with PNI < 47.95 (log-rank *p* = 0.021). In univariable Cox regression analysis, higher PNI was associated with a reduced risk of progression or death (HR 0.37; 95% CI, 0.15–0.90).

In contrast, stratification by the median pre-ASCT PNI value (47.95) revealed that patients with lower PNI exhibited significantly inferior progression-free survival (PFS). The median PFS was 7 months (95% CI, 3.8–10.2) in the low PNI group, compared to 45 months in the high PNI group (PNI ≥ 47.95; log-rank *p* = 0.021). Univariable Cox regression analysis showed that higher pre-ASCT PNI was associated with a significantly reduced risk of disease progression (HR 0.37; 95% CI, 0.15–0.90; *p* = 0.029) (Figure 4). When analyzed as a continuous variable, higher PNI showed a consistent trend toward improved PFS (HR per unit increase 0.92; 95% CI, 0.85–1.00; *p* = 0.058). Detailed univariable Cox regression results are provided in Supplementary Figure S1.

For the multivariable Cox regression model, variables were selected based on univariable analyses (Table 3) and clinical relevance. During follow-up, 22 PFS events were observed among 40 evaluable patients, yielding an events-per-variable ratio of 11 for the two-variable model. In multivariable analysis, both pre-ASCT PNI and treatment response remained independently associated with PFS. Specifically, patients with PNI ≥ 47.95 had a significantly lower risk of disease progression (HR 0.39; 95% CI, 0.16–0.97; *p* = 0.043), and achieving CR prior to ASCT remained significantly associated with reduced progression risk (HR 0.41; 95% CI, 0.17–0.98; *p* = 0.046).

Although patients with higher pre-ASCT PNI tended to favor improved OS, the difference did not reach statistical significance (log-rank *p* = 0.252; univariable Cox HR, 0.47; 95% CI, 0.12–1.77; *p* = 0.265). These findings suggest that PFS may be a more sensitive endpoint than OS for capturing the prognostic impact of host-related factors in the ASCT setting.

## 4. Discussion

In this single-center retrospective study, we evaluated the impact of pre-transplant disease status, nutritional and inflammatory markers, and transplant-related parameters on outcomes in patients with lymphoma undergoing autologous stem cell transplantation (ASCT). The main findings can be summarized as follows: (i) a higher infused CD34^+^ cell dose was associated with improved early transplant-related outcomes, particularly a shorter length of hospitalization; (ii) achieving complete remission prior to ASCT was associated with significantly longer progression-free survival (PFS); (iii) pre-ASCT prognostic nutritional index (PNI) emerged as an independent predictor of PFS; and (iv) although both pre-ASCT response and PNI showed trends toward improved overall survival (OS), these associations did not reach statistical significance, likely due to the limited number of events.

### 4.1. CD34^+^ Cell Dose and Early Transplant Outcomes

The median neutrophil (12 days) and platelet (14 days) engraftment times observed in our cohort are concordant with previously published data in comparable patient populations undergoing autologous hematopoietic stem cell transplantation (AHSCT), where neutrophil and platelet recovery typically occur within 10–14 days and 12–16 days, respectively [17,21,22].

Notably, a higher infused CD34^+^ cell dose (≥3.6 ×10^6^/kg) was associated with a statistically significant reduction in the duration of hospitalization (*p* = 0.010). Prior studies have similarly linked higher CD34^+^ cell doses to accelerated hematopoietic recovery and decreased demand for supportive care—such as reduced transfusion requirements and fewer days of intravenous antibiotic therapy—thereby shortening the length of stay in hospital [18,19,20].

Conversely, the lack of a statistically significant association between CD34^+^ cell dose and neutrophil or platelet engraftment kinetics in our analysis suggests that, beyond a critical threshold, further escalation of cell dose yields diminishing returns regarding recovery kinetics. This observation aligns with the ‘plateau effect’ initially described by Weaver et al., indicating that engraftment kinetics do not necessarily improve proportionally once a minimum effective cell dose is surpassed [17]. These findings underscore that hematopoietic recovery is influenced not only by stem cell quantity but also by additional factors such as prior treatment exposure, marrow reserve, and the inflammatory milieu. Consequently, the clinical relevance of higher CD34^+^ cell doses may be more pronounced in post-engraftment recovery and healthcare resource utilization rather than in engraftment timing per se, a hypothesis that warrants validation in larger, well-powered cohorts.

### 4.2. Pre-ASCT Response and Survival Outcomes

Pre-transplant disease status is a well-recognized determinant of post-autologous stem cell transplantation outcomes in patients with lymphoma. In our cohort, patients transplanted in complete remission (CR) experienced a markedly longer progression-free survival (PFS) compared with those transplanted in partial remission. This finding supports the concept that residual disease burden at the time of transplantation plays a critical role in post-transplant disease control. Achieving a deep remission prior to ASCT likely reflects both chemosensitivity of the lymphoma and a lower burden of clonally resistant tumor cells, thereby reducing the risk of early relapse.

Previous studies have consistently demonstrated that patients achieving complete radiologic or metabolic remission before ASCT have superior outcomes compared with those with residual disease. In relapsed or refractory diffuse large B-cell lymphoma (DLBCL), several retrospective and prospective analyses have shown that pre-ASCT response status is strongly associated with post-transplant failure-free survival and PFS, with patients in CR deriving the greatest benefit from ASCT [1,2]. Moreover, positron emission tomography–based response assessment prior to ASCT has been shown to further refine risk stratification, with PET-negative patients demonstrating significantly improved post-transplant outcomes compared with PET-positive patients, particularly in aggressive lymphoma subtypes [7,23]. Our results are consistent with these observations and extend them to a heterogeneous real-world lymphoma cohort, reinforcing the importance of achieving optimal disease control prior to transplantation. Notably, the prognostic impact of pre-ASCT CR on PFS remained clinically meaningful in our study despite differences in histologic subtypes and prior treatment exposures.

Although patients transplanted in CR also exhibited numerically improved overall survival (OS) in our cohort, this difference did not reach statistical significance. This observation is not unexpected and aligns with previous ASCT studies where improvements in PFS do not invariably translate into statistically significant OS benefits. Potential explanations include the relatively limited sample size, the low number of death events, and the availability of effective post-relapse salvage therapies, which may mitigate OS differences despite clear separation of PFS curves [2,24]. Therefore, PFS may represent a more sensitive endpoint for capturing the prognostic impact of pre-transplant disease status in ASCT-treated patients.

### 4.3. Prognostic Nutritional Index and Survival Outcomes

One of the most important findings of the present study is the identification of pre-ASCT prognostic nutritional index (PNI) as an independent predictor of progression-free survival, even after adjustment for pre-transplant treatment response (HR, 0.39; 95% CI, 0.16–0.97; *p* = 0.043). This observation underscores the critical relevance of host-related biological factors, alongside disease-specific characteristics, in shaping post-transplant outcomes. Consistent with this finding, analysis of PNI as a continuous variable demonstrated a similar trend toward improved PFS with increasing PNI values, supporting a dose–response relationship rather than a strict threshold effect.

PNI reflects both nutritional status and immune competence through its components—serum albumin level and absolute lymphocyte count—and may therefore capture key biological processes that influence post-transplant disease control. Hypoalbuminemia is closely linked to systemic inflammation and activation of pro-inflammatory cytokine pathways, which have been shown to promote tumor progression, impair tissue repair, and modulate the tumor microenvironment in favor of immune escape [8,9]. Similarly, lymphopenia reflects compromised cellular immunity, particularly deficiencies in T-cell-mediated antitumor responses, which have been linked to reduced immune surveillance and increased risk of disease recurrence across multiple malignancies [10,11]. In the context of high-dose chemotherapy and autologous stem cell transplantation, these factors may adversely affect immune reconstitution, tolerance to transplant-related toxicity, and the ability to control minimal residual disease.

The prognostic relevance of PNI has been demonstrated across a wide spectrum of malignancies. Initially characterized by Onodera et al. [12] as a predictor of surgical outcomes, PNI has since been associated with survival in both solid and hematologic malignancies, including lymphomas treated with conventional chemotherapy. In the hematopoietic stem cell transplantation (HSCT) setting, lower pre-transplant PNI has been linked to increased transplant-related complications, higher non-relapse mortality, and inferior survival, particularly in allogeneic transplantation cohorts [12,13]. While data specifically regarding the autologous setting remain sparse, our findings extend the existing literature by demonstrating the independent prognostic value of pre-ASCT PNI for progression-free survival (PFS) in lymphoma patients.

Notably, the prognostic utility of PNI in our cohort appears to be complementary to established disease-related indices, such as the International Prognostic Index (IPI). While IPI primarily reflects tumor burden and baseline disease aggressiveness at diagnosis [6], PNI captures the patient’s physiological reserve, nutritional status, and immune capacity at a time point closer to transplantation [6]. Interestingly, BMI alone was not significantly associated with PFS in our study, suggesting that simple anthropometric measures may be insufficient to capture the complex interaction between nutritional status, immune competence, and post-transplant outcomes. This finding aligns with the concept of ‘sarcopenic obesity’ in hemato-oncology, where body mass may mask underlying physiological frailty [25]. The persistence of PNI as an independent factor in our multivariable analysis—even after accounting for treatment response and BMI—underscores the distinct and clinically meaningful role of host resilience in post-transplant disease control.

Conversely, while higher pre-ASCT PNI was associated with a numerical improvement in overall survival, this association did not reach statistical significance. This pattern, frequently observed in transplant studies, may reflect limited statistical power, the low frequency of mortality events, and the confounding influence of effective post-relapse salvage therapies [14,15]. Taken together, our results suggest that PNI may be more closely linked to immediate disease control and progression risk than to long-term survival endpoints.

From a clinical perspective, identifying PNI as a prognostic marker has implications beyond risk stratification. Unlike many disease-related factors, nutritional and immunological status may represent modifiable targets. Previous studies in hematologic malignancies and stem cell transplantation settings have suggested that poor pre-transplant nutritional status is associated with increased complications and inferior outcomes [16]. Although prospective data are lacking, our findings provide a rationale for future studies evaluating whether targeted nutritional support or immunomodulatory interventions in patients with low pre-ASCT PNI can improve transplant tolerance and reduce early progression risk. Consequently, PNI may serve not only as a prognostic biomarker but also as a practical tool to guide individualized supportive care strategies in patients undergoing ASCT.

### 4.4. Transplant-Related Complications and Safety

The rates of transplant-related complications observed in our cohort were comparable to those reported in the literature for patients undergoing autologous stem cell transplantation with BEAM conditioning. Febrile neutropenia and documented infections occurred at frequencies consistent with previously published series, where infectious complications remain prevalent despite significant advances in supportive care. Notably, no cases of veno-occlusive disease (VOD) were recorded, aligning with reports of a low VOD incidence in the autologous setting, particularly following BEAM-based regimens [24,26].

Furthermore, both intensive care unit admission and 100-day transplant-related mortality rates were low in our cohort. Reported 100-day TRM rates following ASCT for lymphoma typically range between 2% and 5% in contemporary series, reflecting improvements in patient selection, conditioning regimens, and supportive care strategies. The observed TRM rate in our study falls within this expected range, further supporting the safety of the procedure in our center. Similarly, the low requirement for ICU admission suggests effective early complication management and appropriate peri-transplant monitoring. [3,24]

Collectively, these findings indicate that ASCT was generally well tolerated in our cohort, with minimal early transplant-related morbidity and mortality. This favorable safety profile likely reflects strict adherence to established transplant protocols, careful patient selection, and optimized supportive care. These results support the feasibility and safety of ASCT in real-world practice, even in a heterogeneous lymphoma population treated at experienced centers.

Our study adds to the growing body of literature on the prognostic role of nutritional status in lymphoma by specifically focusing on patients undergoing autologous stem cell transplantation—a setting less extensively studied compared to allogeneic transplantation. Importantly, the integration of host-related factors such as PNI with disease-related response status (CR vs. PR) provides a more comprehensive framework for pre-transplant risk stratification.

From a clinical perspective, PNI represents a simple, readily available, and potentially modifiable parameter that may be incorporated into routine pre-transplant evaluation. However, given the relatively small sample size and retrospective design, these findings should be considered exploratory and hypothesis-generating. This is consistent with prior studies demonstrating the prognostic value of nutritional and inflammation-based indices in lymphoma and cancer populations [15]. Identification of patients with low PNI could facilitate targeted interventions, including nutritional optimization, physical prehabilitation, and closer monitoring during the peri-transplant period.

Taken together, these findings suggest that integrating host-related and disease-related factors may enhance risk stratification and inform personalized management strategies in lymphoma patients undergoing ASCT.

### 4.5. Limitations

Several limitations of this study should be acknowledged. First, the retrospective, single-center design may limit the generalizability of the findings. Second, the relatively small sample size and limited number of survival events reduced the statistical power, particularly for subgroup analyses and overall survival endpoints, and may increase the risk of model overfitting in multivariable analyses. While our cohort included multiple lymphoma subtypes, detailed subtype-specific analyses were not feasible. Exploratory comparisons between Hodgkin and non-Hodgkin lymphoma yielded no significant differences in PFS; however, these results should be interpreted with caution, as the impact of histologic heterogeneity cannot be entirely excluded. Furthermore, the International Prognostic Score (IPS) for Hodgkin lymphoma was not included because its component variables were not uniformly recorded at the same clinical time point in this retrospective cohort. Specifically, disease stage was documented at diagnosis, whereas ECOG performance status and laboratory parameters were assessed prior to transplantation, precluding reliable and standardized IPS calculation. In addition, the use of a median-based PNI cut-off may limit comparability and external applicability, as previously reported thresholds vary across studies, and no standardized value has been established. This was partially addressed by complementary analyses treating PNI as a continuous variable. Due to the limited sample size and number of events, data-driven cut-off optimization methods such as ROC- or quartile-based analyses were not pursued to avoid overfitting, instability of threshold estimates, and inflation of type I error. Therefore, the identified cut-off should be considered exploratory and hypothesis-generating.

Due to the limited number of events, the multivariable model was restricted to two covariates to minimize the risk of overfitting. Therefore, residual confounding from unmeasured or incompletely captured variables—such as comorbidities, performance status, and infection-related factors—cannot be excluded. In addition, important disease-related factors such as IPI score, LDH levels, disease stage, and lymphoma subtype may also contribute to residual confounding and were not included in the multivariable model due to sample size constraints. Furthermore, additional sensitivity analyses adjusting for multiple clinical covariates were not feasible due to sample size constraints. Consequently, the findings should be interpreted as hypothesis-generating rather than definitive and require validation in larger, prospective, and multicenter cohorts. Future studies should also explore the integration of PNI into multivariable prognostic models incorporating PET-based response assessment and molecular features.

## 5. Conclusions

In conclusion, this single-center retrospective study suggests that both disease-related and host-related factors play complementary roles in determining outcomes following autologous stem cell transplantation (ASCT) in patients with lymphoma. Achieving complete remission prior to ASCT and a higher pre-transplant prognostic nutritional index were associated with improved progression-free survival. Notably, PNI remained an independent predictor of outcomes even after adjusting for treatment response and body mass index. These findings underscore the clinical necessity of dynamic pre-transplant assessments that extend beyond traditional baseline prognostic scores and simple anthropometric measures.

Given its simplicity and accessibility, PNI may serve as a practical and cost-effective tool for risk stratification and patient optimization in the transplant setting. While our results should be considered exploratory and hypothesis-generating due to the modest sample size, they provide a strong rationale for prospective multicenter studies. Such research is warranted to validate these findings and to investigate whether targeted nutritional support or prehabilitative interventions can further improve transplant outcomes. Incorporating PNI into routine pre-transplant evaluations may facilitate the identification of vulnerable patients who could benefit from early supportive care and intensified post-transplant surveillance.

## Figures and Tables

**Table 1:** Baseline characteristics of the study population.

Variable	Total (*n* = 43)
Age, median (IQR), years	38 (IQR 29)
Sex, *n* (%)	
Male	31 (72.1)
Female	12 (27.9)
ECOG	
0	32 (74.4)
1	11 (26.6)
BMI, kg/m^2^, median (IQR)	26 (IQR 5)
BMI category, *n* (%)	
<25 kg/m^2^	19 (44.2)
≥25 kg/m^2^	24 (55.8)
Histology, *n* (%)	
Diffuse large B-cell lymphoma	17 (39.5)
Hodgkin lymphoma	19 (44.2)
Mantle cell lymphoma	5 (11.6)
T-cell lymphoma	2 (4.7)
IPI score (DLBCL only, *n* = 17), *n* (%)	
Low	4 (24)
Intermediate	6 (35)
High	7 (41)
Prior lines of therapy, *n* (%)	
1–2 lines	38 (88.4)
≥3 lines	5 (11.6)
Pre-ASCT response, *n* (%)	
CR	23 (53.5)
Non-CR	20 (46.5)
Pre-ASCT LDH, *n* (%)	
Normal	23 (53.5)
High	20 (46.5)
Serum albumin, g/dL, median (IQR)	4.2 (IQR 0.4)
ALC, /mm^3^, median (IQR)	1000 (IQR 700)
PNI (cut-off: 47.95), *n* (%)	
Low (<47.95)	22 (51.2)
High (≥47.95)	21 (48.8)
Conditioning regimen, *n* (%)	
BEAM	39 (90.7)
Other	4 (9.3)
Follow-up duration after ASCT, months	48 (IQR 18)

Abbreviations: IQR, interquartile range; ASCT, autologous stem cell transplantation; IPI, International Prognostic Index; CR, complete remission; LDH, lactate dehydrogenase; ALC, absolute lymphocyte count (/mm^3^); PNI, prognostic nutritional index; BEAM, carmustine, etoposide, cytarabine, and melphalan.

**Table 2:** Early transplant-related outcomes after autologous stem cell transplantation.

Variable	Value, n (%) or Median (IQR)
Infused CD34^+^ cell dose (×10^6^/kg)	3.6 (IQR 2.5)
Neutrophil engraftment achieved *	42 (97.7)
Time to neutrophil engraftment, days *	12 (IQR 1)
Platelet engraftment achieved	42 (97.7)
Time to platelet engraftment, days	14 (IQR 2.25)
Length of hospitalization, days	28 (IQR 9)
Febrile neutropenia within 100 days	40 (93.0)
Documented infection within 100 days	17 (39.5)
ICU admission	2 (4.7)
100-day TRM	2 (4.7)

* Neutrophil engraftment was defined as an absolute neutrophil count ≥500/µL for three consecutive days. Platelet engraftment was defined as a platelet count ≥20,000/µL for seven consecutive days without transfusion support. Values are presented as median (interquartile range) or number (%), as appropriate. Abbreviations: IQR, interquartile range; ICU, intensive care unit; TRM, transplant-related mortality; CD34^+^, cluster of differentiation 34–positive cells.

**Table 3:** Univariable Cox proportional hazards regression analysis for progression-free survival.

Variable	HR	95% CI	*p*-Value
Age (per year increase)	1.009	0.98–1.04	0.57
Sex (male vs. female)	1.183	0.48–2.91	0.72
Histology (HL vs. NHL)	1.15	0.49–2.72	0.75
IPI score	0.69	0.25–1.90	0.47
Pre-ASCT response (CR vs. PR)	0.38	0.18–0.82	0.03 *
CD34 dose (<3.6 vs. ≥3.6 ×10^6^/kg)	0.54	0.22–1.29	0.16
BMI (≥25 vs. <25 kg/m^2^)	0.79	0.34–1.81	0.57
PNI (continuous, per unit increase)	0.92	0.85–1.00	0.062
PNI (≥47.95 vs. <47.95)	0.37	0.15–0.90	0.029 *

Abbreviations: HR, hazard ratio; CI, confidence interval; HL, Hodgkin lymphoma; NHL, non-Hodgkin lymphoma; ASCT, autologous stem cell transplantation; PNI, prognostic nutritional index; BMI, body mass index. PNI was evaluated both as a continuous variable (per unit increase) and as a categorical variable using the median cut-off value (47.95) in univariable analyses. Only one PNI parameter was included in multivariable models to avoid collinearity. Variables with clinical relevance and/or *p* < 0.10 in univariable analysis were considered for inclusion in multivariable Cox regression models. * Statistically significant (*p* < 0.05).

## Data Availability

The datasets generated and/or analyzed during the current study are available from the corresponding author on reasonable request.

## Supplementary Materials

The following supporting information can be downloaded at: https://www.mdpi.com/article/10.3390/jcm15093549/s1. Figure S1. Univariable Cox regression analysis of progression-free survival.
