# Supplementary material for: Pre-Transplant Prognostic Nutritional Index Independently Predicts Progression-Free Survival After Autologous Stem Cell Transplantation in Lymphoma
Source: J Clin Med. 2026 May 6;15(9):3549. doi: 10.3390/jcm15093549 (PMC13164050; doi:10.3390/jcm15093549)
Supplement: Supplementary file 1 [file jcm-15-03549-s001.zip › jcm-4255974-supplementary.pdf]

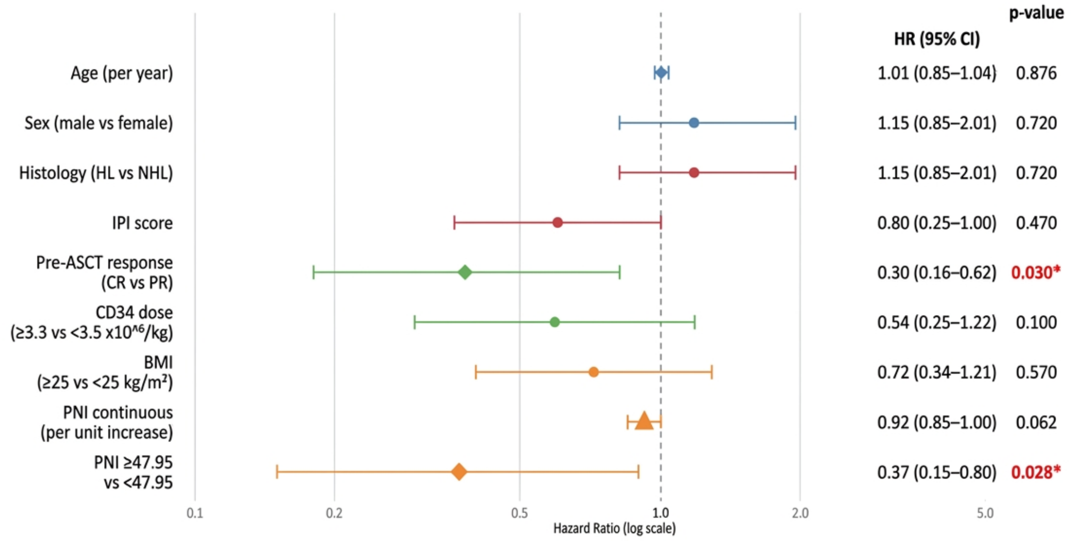

Figure S1. Univariable Cox regression analysis of progression-free survival. Forest plot showing hazard ratios (HRs) with 95% confidence intervals for clinical and laboratory variables. Pre-transplant response (CR vs PR) and PNI (≥47.95 vs <47.95) were significantly associated with PFS.
